# Auxin biosynthesis maintains embryo identity and growth during BABY BOOM-induced somatic embryogenesis

**DOI:** 10.1093/plphys/kiab558

**Published:** 2021-12-01

**Authors:** Mengfan Li, Justyna Wrobel-Marek, Iris Heidmann, Anneke Horstman, Baojian Chen, Ricardo Reis, Gerco C Angenent, Kim Boutilier

**Affiliations:** 1 Bioscience, Wageningen University and Research, Wageningen, 6700 AA, Netherlands; 2 Laboratory of Molecular Biology, Wageningen University and Research, Wageningen, 6700 AP, Netherlands; 3 Faculty of Natural Sciences, Institute of Biology, Biotechnology and Environmental Protection, University of Silesia in Katowice, Katowice, 40-032, Poland; 4 Enza Zaden Research and Development B.V, Enkhuizen, 1602 DB, The Netherlands

## Abstract

Somatic embryogenesis is a type of plant cell totipotency where embryos develop from nonreproductive (vegetative) cells without fertilization. Somatic embryogenesis can be induced in vitro by auxins, and by ectopic expression of embryo-expressed transcription factors like the BABY BOOM (BBM) AINTEGUMENTA-LIKE APETALA2/ETHYLENE RESPONSE FACTOR domain protein. These different pathways are thought to converge to promote auxin response and biosynthesis, but the specific roles of the endogenous auxin pathway in somatic embryogenesis induction have not been well-characterized. Here we show that BBM transcriptionally regulates the *YUCCA3* (*YUC3*) and *YUC8* auxin biosynthesis genes during BBM-mediated somatic embryogenesis in Arabidopsis (*Arabidopsis thaliana*) seedlings. BBM induced local and ectopic *YUC3* and *YUC8* expression in seedlings, which coincided with increased *DR5* auxin response and indole-3-acetic acid (IAA) biosynthesis and with ectopic expression of the *WOX2* embryo reporter. YUC-driven auxin biosynthesis was required for BBM-mediated somatic embryogenesis, as the number of embryogenic explants was reduced by ca. 50% in *yuc3 yuc8* mutants and abolished after chemical inhibition of YUC enzyme activity. However, a detailed YUC inhibitor time-course study revealed that YUC-dependent IAA biosynthesis is not required for the re-initiation of totipotent cell identity in seedlings. Rather, YUC enzymes are required later in somatic embryo development for the maintenance of embryo identity and growth. This study resolves a long-standing question about the role of endogenous auxin biosynthesis in transcription factor-mediated somatic embryogenesis and also provides an experimental framework for understanding the role of endogenous auxin biosynthesis in other in planta and in vitro embryogenesis systems.

## Teaser

The BABY BOOM transcription factor induces YUCCA-dependent auxin biosynthesis during somatic embryogenesis to maintain embryo identity and development.

## Introduction

Totipotency is the capacity of a single cell to regenerate into a complete organism (Condic, 2014). Totipotency is restricted to the zygote in sexually reproducing plants, but some asexually reproducing plants also produce embryos from vegetative cells and from unfertilized gametes (Pichot et al., 2001; Garcês et al., 2007; Schmidt, 2020). Induced totipotency refers to the ability of cells to develop into embryos when cultured in vitro (Fehér, 2019). Somatic embryogenesis is a type of totipotency in which vegetative (nongametophytic) cells are induced to develop into embryos after exposure to exogenous growth regulators, in particular the synthetic auxin 2,4-dichlorophenoxy acetic acid (2,4-D), or by ectopic expression of embryo or meristem identity transcription factors (Horstman et al., 2017a; Fehér, 2019; Karami et al., 2021b). Both inducer treatments promote cell division and also reprogram cells in a multicellular explant toward somatic embryogenesis or toward pluripotent pathways resulting in callus formation and organogenesis. How both 2,4-D and transcription factors induce a subset of cells in an explant to develop specifically into somatic embryos is not known, but roles for chromatin modifications as well as for changes in expression of embryo identity genes and plant growth regulator pathway genes have been proposed (De-la-Peña et al., 2015; Horstman et al., 2017a; Wang et al., 2020; Wójcik et al., 2020).

2,4-D efficiently induces somatic embryogenesis in a wide range of explants in the model plant Arabidopsis (*Arabidopsis thaliana*). As in other plants, Arabidopsis somatic embryos either develop directly from the explant (Luo and Koop, 1997; Gaj, 2001; Kobayashi et al., 2010) or indirectly from embryogenic callus (Ikeda-Iwai et al., 2003; Su et al., 2009). In the direct system, fully differentiated embryos with root and shoot meristems and cotyledons develop in the presence of 2,4-D, while in the indirect system removal of 2,4-D from the culture medium is usually required to promote differentiation (patterning) of pro-embryogenic masses, which are multicellular embryos lacking radial and apical–basal patterning (Halperin and Jensen, 1967; Gaj, 2011). Ectopic expression of specific embryo or meristem identity transcription factors also induces somatic embryo formation, but can do so in the absence of exogenous plant growth regulators (Horstman et al., 2017a). Among these are the LEAFY COTYLEDON 1 (LEC1) HAP3/CCAAT binding protein, the LEC2 B3-domain protein, and the BABY BOOM (BBM) clade of AINTEGUMENTA-LIKE (AIL) APETALA2/ETHYLENE RESPONSE FACTOR (AP2/ERF) transcription factors, which also includes the PLETHORA (PLT) proteins (Lotan et al., 1998; Stone et al., 2001; Gaj et al., 2005; Horstman et al., 2017b). Ectopic over-expression of these transcription factors in germinating seeds induces direct somatic embryo formation on above-ground organs of seedlings, including the cotyledon petioles, tip and margin and the shoot apical meristem. The mechanisms driving transcription factor-induced somatic embryogenesis have not been well-studied, but like 2,4-D-induced somatic embryogenesis, are thought to require chromatin-level changes as well as deregulation of embryo/meristem identity transcription factor and auxin pathway genes (Horstman et al., 2017a; Tian et al., 2020; Wójcik et al., 2020).

Transcriptional activation of auxin biosynthesis genes is one of the common regulatory points downstream of 2,4-D and transcription factor-induced somatic embryogenesis. Plants synthesize auxin by different pathways (Normanly, 2010; Zhao, 2014). The major auxin in Arabidopsis is indole-3-acetic acid (IAA), which is mainly synthesized through the TRYPTOPHAN AMINOTRANSFERASE ARABIDOPSIS (TAA)/YUCCA (YUC) pathway (Zhao, 2014). Enzymatic activity of (TAA1 and TAA1-RELATED PROTEINS (TARs) convert TRP into the intermediate product indole-3-pyruvic acid (IPyA), which is then converted into IAA by the YUC flavin-dependent monooxygenases (Stepanova et al., 2011). The Arabidopsis genome contains three *TAA1/TAR* genes and 11 *YUC* monooxygenase genes that are differentially expressed during plant development (Cheng et al., 2006, 2007; Wang et al., 2011; Hentrich et al., 2013; Robert et al., 2013). Arabidopsis TAA/TARs and YUC proteins each function in a redundant manner, such that many of their functions only become evident in higher-order mutant combinations (Cheng et al., 2006, 2007; Wang et al., 2011; Robert et al., 2013).

Endogenous auxin, mainly IAA, is often elevated in cells or tissues undergoing 2,4-D-induced somatic embryogenesis (Michalczuk et al., 1992; Charrière et al., 1999; Pasternak et al., 2002). In the Arabidopsis direct somatic embryogenesis system, exposure of immature zygotic embryo explants to 2,4-D induces expression of *YUC1* and *YUC4* early in somatic embryogenesis, followed later by *TAA1* and *YUC10* expression (Wójcikowska et al., 2013). Single *yuc* mutants have no obvious phenotype under normal growth conditions, except the *yuc8-1* mutant, which shows reduced seed set (Cheng et al., 2006, 2007; Ståldal et al., 2012). However, in 2,4-D-induced somatic embryo cultures, single *yuc2* and *yuc4* mutants produce fewer embryogenic explants and fewer somatic embryos per explant compared to wild-type (WT) explants (Wójcikowska et al., 2013). In the indirect somatic embryogenesis system, where embryos develop after an initial callus phase, *YUC* gene expression *(YUC1, YUC2, YUC4*, and *YUC6*) is detected late in the development of embryogenic callus and then increases after transfer of the callus to 2,4-D-free medium (Bai et al., 2013). In this system, the quadruple *yuc1 yuc2 yuc4 yuc6* mutant shows a normal progression of somatic embryogenesis, while the *yuc1 yuc4 yuc10 yuc11* mutant produces only a few malformed somatic embryos (Bai et al., 2013). Treatment with the YUC enzyme inhibitor yucasin drastically reduces somatic embryo formation from *Coffea canephora* explants (Uc-Chuc et al., 2020). It is clear that endogenous auxin biosynthesis has a role in 2,4-D-induced somatic embryo induction, but when and how auxin biosynthesis specifically promotes somatic embryogenesis is not known.

LEC and BBM/PLT transcription factors have also been shown to bind to and/or transcriptionally regulate auxin biosynthesis genes during normal plant development and under conditions that promote somatic embryo development. Ectopic LEC2 expression induces *YUC2* and *YUC4* expression early during somatic embryo development from seedlings (Stone et al., 2008), and ectopic LEC1 expression induces *YUC* gene expression during 2,4-D-induced somatic embryogenesis from immature zygotic embryos (*YUC1*, *YUC4*, and *YUC10*) and from seedlings (*YUC10*; Junker et al., 2012; Wójcikowska et al., 2013). CHOTTO1/EMBRYOMAKER/PLT5/AIL5 binds to and transcriptionally regulates *YUC4* in the shoot apex (Pinon et al., 2013), while PLT2/AIL4 binds to and transcriptionally regulates *YUC3* and *YUC8* in the root tip (Santuari et al., 2016). BBM/AIL2 also binds to *YUC3* and *YUC8* during 2,4-D- and BBM-induced somatic embryogenesis, but it is not known if BBM also transcriptionally regulates these genes (Horstman et al., 2017b). Although auxin biosynthesis genes are downstream targets of embryo identity transcription factors during somatic embryogenesis, it is not known whether auxin biosynthesis is required to promote transcription factor-driven somatic embryogenesis.

Here we examined the role of YUC-dependent IAA biosynthesis in BBM-induced somatic embryogenesis from Arabidopsis seedling cotyledons. Using a combination of genetic analysis, pharmacological inhibition and cell fate analysis we show that YUC-dependent IAA biosynthesis is essential for BBM-mediated somatic embryogenesis, but that this pathway is only required after the initiation of totipotency, for the subsequent proliferation and differentiation of embryogenic cells.

## Results

### Developmental steps in *BBM*-induced somatic embryogenesis

**Figure 1:**
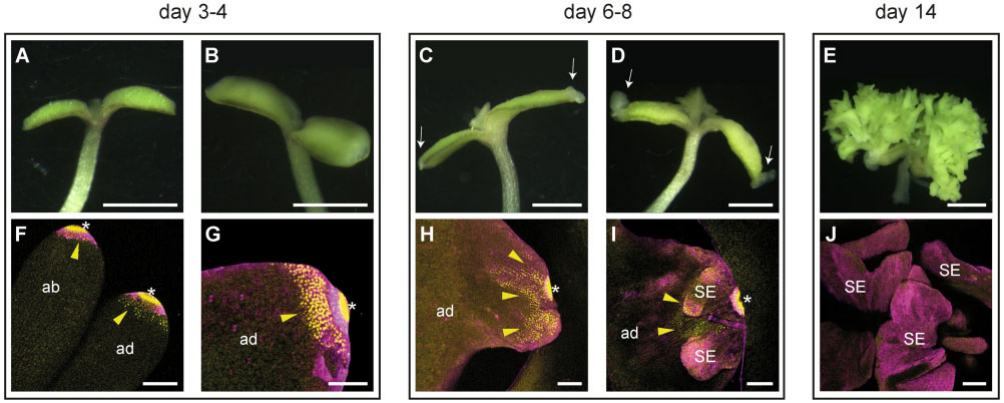
Overview of BBM-induced somatic embryogenesis. A–E, Light micrographs of representative DEX-treated *35S:BBM-GR* explants. F–J, Confocal laser scanning micrographs of WOX2:YFP expression at the cotyledon tip of DEX-treated *35S:BBM-GR* explants. The day of culture is indicated above the images. Arrowheads, WOX2-YFP expression. Arrows, growth protrusions. Asterisks, autofluorescence. ad, adaxial side. ab, abaxial side. SE, somatic embryo. Scale bars: A–E, 1 mm; F–J, 100 µm.

The normal course of somatic embryogenesis in seedlings from dexamethasone (DEX)-treated *35S:BBM-GR* seeds has been described previously (Horstman et al., 2017b; Godel-Jedrychowska et al., 2020) and is summarized in Figure 1. DEX treatment induces posttranslational nuclear localization of the BBM-GR fusion protein (Horstman et al., 2017b), allowing comparison of samples with and without ectopic BBM activity. Embryogenic cell divisions are observed in the cotyledons of DEX-treated *35S:BBM-GR* seedlings around Days 3–4 of culture (Figure 1, A and B). These divisions begin at the cotyledon tip, followed by the cotyledon margin and shoot apex and are visualized as thickened, smooth, and light green tissue. By Days 6–8 of culture small embryogenic protrusions can be observed on the dividing tip (Figure 1, C and D) and by Day 14 a mass of primary and secondary somatic embryos develops on the seedling cotyledon (Figure 1E).

Previously we showed that the embryo identity and BBM direct target gene *LEC1* is expressed on the cotyledon tip of DEX-treated *35S:BBM-GR* seeds as early as 1 d after DEX treatment and becomes more highly expressed at the cotyledon tip and margin when these tissues begin to proliferate (Horstman et al., 2017b). We followed the expression of the *WOX2:NLS-3xYFP* embryo marker to determine whether embryo identity genes that are not direct BBM targets are expressed in the same way. During the first 2 d of culture *WOX2:NLS-3xYFP* expression was detected in both control (mock-treated) and DEX-treated seedlings throughout the seedling, and in the cotyledon on the abaxial and adaxial surface (Supplemental Figure S1, A and B; Figure 8). The nuclear WOX2-YFP signal could no longer be detected in the control seedling cotyledons from Day 3 onward (Supplemental Figure S1C), but was maintained and became restricted to the tip of the cotyledon in the DEX-treated seedlings (Figure 1, F and G). During Days 6–8 of culture, WOX2-YFP expression was observed on the explant in the region where embryos develop and in the embryogenic growths of most DEX-treated control seedlings (Figure 1, H and I). In the *35S: BBM-GR* line used in this study, 10%–15% of the seedlings do not form somatic embryos and the same proportion of seedlings lacked WOX2-YFP expression in the cotyledon (Supplemental Figure 1D). By Day 14 of culture WOX2-YFP expression could only be detected in ca. 20% of these embryos (Supplemental Table S1).

The above data indicate that expression of the BBM direct target gene *LEC1* precedes expression of the nontarget gene *WOX2*. Both *LEC1* and *WOX2* are initially expressed on the cotyledon tip, the site where somatic embryo formation is first initiated. LEC1 is a major regulator of early and late embryo development pathways and overexpression of LEC1 induces spontaneous somatic embryogenesis. LEC1 also acts a pioneer factor at the *FLOWERING LOCUS C* gene by promoting an active chromatin state (Tao et al., 2017). Activation of *LEC1* expression by BBM might therefore be required for promoting chromatin accessibility at BBM target loci and/or for parallel activation of early embryo development genes.

### BBM regulates auxin pathway genes

**Figure 2:**
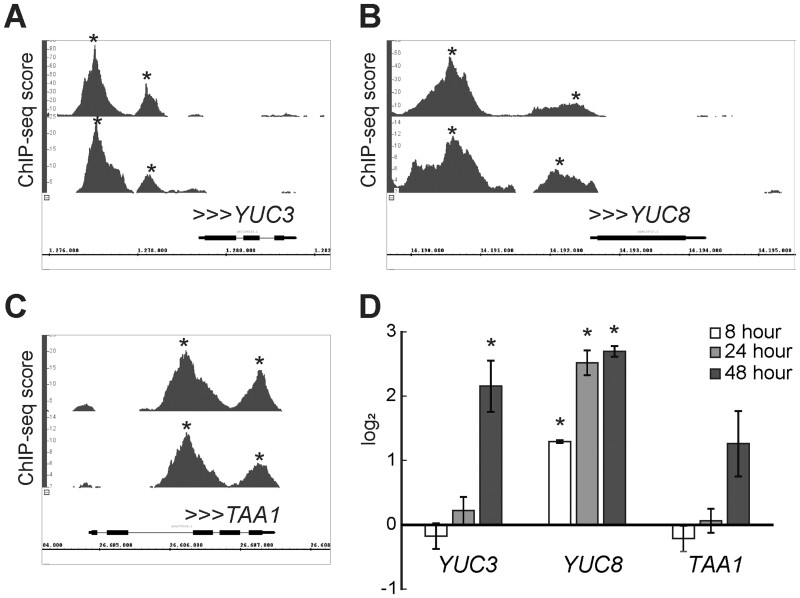
BBM binds and regulates the expression of auxin biosynthesis genes. A–C, ChIP-seq BBM binding profiles for auxin biosynthesis genes in somatic embryo tissue. The binding profiles for *35S::BBM-GFP* (upper profile) and *BBM::BBM-YFP* (lower profile) are shown. The *x*-axis shows the nucleotide position of DNA binding in the selected genes (TAIR 10 annotation), the y-axis shows the ChIP-seq score, and the arrowheads indicate the direction of gene transcription. Peaks with scores above 1.76 for *35S::BBM-GFP* and 3.96 for *pBBM::BBM-YFP* were considered statistically significant (*, false discovery rate < 0.05). The ChIP-seq data were generated in Horstman et al. (2015). The ChIP-seq data and data analysis can be downloaded from GEO (GSE52400). The plots were generated using Integrated Genome Browser. D, The relative expression of auxin biosynthesis genes during seed germination was determined by qPCR for DEX-treated *35S::BBM-GR* seedlings using mock-treated Col-0 seeds as the calibrator and the *SAND* gene (Czechowski et al., 2005) as the reference. Error bars indicate standard errors of the three biological replicates in the same genetic background. Asterisk, statistically significant change in gene expression levels, determined using Student’s t-test (*P* < 0.05).

The BBM transcription factor binds a number of key regulatory genes during 2,4-D and BBM-induced somatic embryogenesis, including genes that promote in vitro regeneration and meristem identity and proliferation (Supplemental Data Set S1; Horstman et al., 2015; Horstman et al., 2017b). Among the direct BBM gene targets are also a number of auxin pathway genes, including the *YUC3*, *YUC8*, and *TAA1* auxin biosynthesis genes. The BBM-binding sites at these loci are shown in Figure 2, A–C. To determine whether BBM also transcriptionally regulates these genes, we analyzed their expression using reverse transcription quantitative PCR (RT-qPCR) in DEX-treated *35S:BBM-GR* seeds at 8, 24, and 48 h after imbibition (pregermination). *YUC3* and *YUC8* expression was significantly upregulated in DEX-treated *35S:BBM-GR* seeds compared to DEX-treated WT seeds, with *YUC3* expression (48 h) lagging behind that of *YUC8* (8 h), while *TAA1* expression was not significantly regulated (Figure 2D). We therefore focused our efforts on *YUC3* and *YUC8* as candidate early auxin biosynthesis target genes.

**Figure 3:**
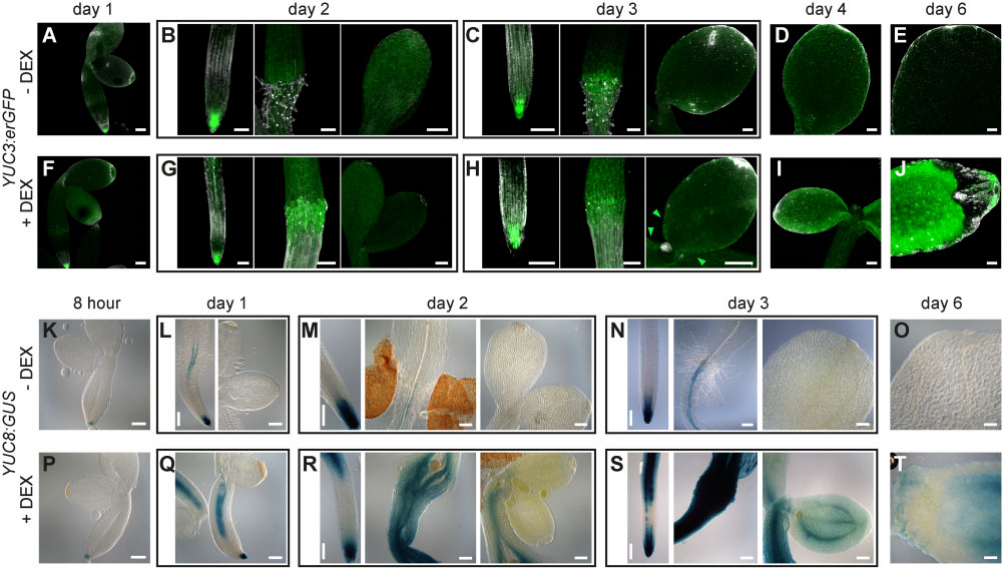
BBM overexpression induces ectopic expression of *YUC3* and *YUC8*. Images of roots, hypocotyls and cotyledons from *YUC* reporter lines in a *35S:BBM-GR* background with (solid gray line) or without (dashed gray line) DEX treatment. The day of culture is shown above the images. A–J, Confocal light scanning micrographs of *YUC3:erGFP* expression. K–T, Light micrographs of *YUC8:GUS* expression. Scale bars, 100 μm.

Next, we examined the spatial and temporal regulation of *YUC3*/*YUC8* expression in *35S:BBM-GR* seeds carrying the *YUC3:erGFP* or the *YUC8:β-glucuronidase* (*GUS*) reporters. Seeds were imbibed and then cultured with or without 10 µM DEX. In WT Arabidopsis seedlings, *YUC3* is expressed in the root meristem and root–hypocotyl transition zone and *YUC8* is expressed in the root vascular tissue and meristem (Ståldal et al., 2012; Chen et al., 2014; Santuari et al., 2016; Figure 3) BBM-enhanced *YUC3* expression was observed in the root–hypocotyl transition zone from Day 2 of culture (Figure 3, B, C, G, and H), followed by weak, but consistent ectopic expression on the proximal cotyledon margin on Day 3 (Figure 3, C and H) and the entire cotyledon surface by Day 4 (Figure 3, D, E, I, and J). Enhanced *YUC8* expression in the hypocotyl vascular tissue was observed after 1 d of culture (Figure 3, L and Q), and *35S:BBM*-induced changes in hypocotyl morphology were already visible after 2 d of culture (Figure 3, M and R). Ectopic expression of *YUC8* was observed in the cotyledons starting from Day 3 of culture (Figure 3, N and S). As in the root–hypocotyl, *YUC8* was also expressed in the cotyledon vascular tissue. After 6 d of culture, areas lacking *YUC3* and *YUC8* expression were observed in a region close to the cotyledon tip (Figure 3, J and T), corresponding to the first sites of somatic embryo induction in DEX-treated *35S:BBM-GR* lines (Figure 1, B and C). Notably, expression of a *YUC3:GUS* reporter that lacks the BBM binding site motif and that is not expressed in the root meristem (Chen et al., 2014) did not show altered expression in DEX-treated *35S:BBM-GR* seedlings (Supplemental Figure S2, B and C).

Together these analyses show that BBM transcriptionally regulates *YUC3* and *YUC8* expression early during somatic embryo induction, both in their native expression domain in the root/hypocotyl, as well as ectopically in the cotyledon. Ectopic *YUC* expression in cotyledons also coincided with the onset of ectopic *WOX2* expression (Figure 1G), suggesting a major change in cotyledon cell fate at this time point. BBM-induced *YUC3/YUC8* expression in cotyledons lagged behind *YUC3/YUC8* expression in the root/transition zone. Germination relies mainly on translation of stored mRNAs (Sano et al., 2020), and postgermination light-grown cotyledons only undergo a few cell divisions (Sano et al., 2020), thus de novo BBM-induced transcription in cotyledons might require activation of cell division and/or reprogramming of chromatin to a transcriptionally active state, processes that are already active in the root and hypocotyl.

### BBM enhances auxin response and biosynthesis

The above results indicate that *YUC3* and *YUC8* are transcriptionally regulated by BBM early during somatic embryo induction*.* We therefore investigated whether these changes are reflected in increased auxin response and IAA levels in seedlings.

**Figure 4:**
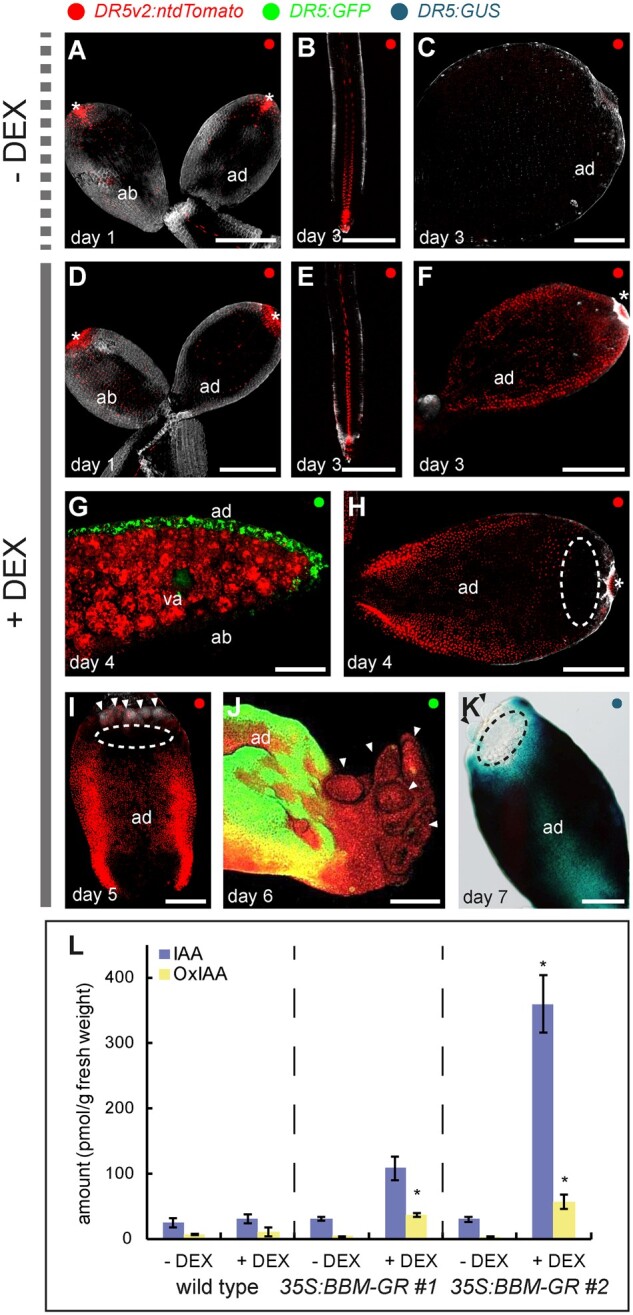
BBM expression enhances *DR5* auxin response and IAA biosynthesis. Confocal laser scanning micrographs of cotyledons or roots from *35S:BBM-GR DR5* seedlings grown without (A–C) and with (D–J) DEX. D–F, H and I are images of *DR5v2:ntdTomato* cotyledons or roots. G and J, These are images of *DR5:GFP* cotyledons. The images in (G) and (J) are counterstained with FM4-64. K, Light image of *DR5:GUS* expression in the cotyledon of a DEX-treated *35S:BBM-GR* seedling. Samples were counter stained with SR2200 (gray, A–F, H and I) or outlined using red autofluorescence (G and J). The dashed ellipses in (H), (I), and (K) indicate the *DR5* minimum. Small embryogenic protrusions are indicated with arrowheads in (I) and (J). va, vascular tissue; asterisks autofluorescence. Scale bars, 200 μm. L, IAA and oxIAA concentrations in seedlings of WT Col-0 and two *35S:BBM-GR* lines grown in the absence or presence of DEX (three technical replicates, each 200 mg). *, samples that showed statistically significant differences in IAA or oxIAA concentrations compared to the non-DEX treated *35S:BBM-GR* control (Student’s t test, *p* < 0.05). Error bars represent the standard deviation of the replicates.

We used *DR5* reporters to follow the temporal and spatial dynamics of auxin response during BBM-mediated somatic embryogenesis. *35S:BBM-GR DR5* seeds were germinated with or without 10 μM DEX and *DR5* expression followed in the explants for 7 d (Figure 4). Weak *DR5* expression was observed on the adaxial and abaxial surfaces of cotyledons (Figure 4, A and D) of both DEX-treated and control seedlings after 1 d of culture. From Day 3 of culture onward, *DR5* expression in the vascular tissue extended further into the root elongation zone in DEX-treated seedlings than in control seedlings (Figure 4, B and E). At this time, *DR5* expression was no longer visible in control cotyledons, but broadened and increased in intensity on the adaxial surface of cotyledons from DEX-treated samples (Figure 4, C and F), where it localized to the adaxial epidermal/subepidermal layers and the vascular bundles (Figure 4G). In the following days, *DR5* expression continued to increase in DEX-treated seedlings, especially along the cotyledon margin (Figure 4, H and I). Starting around Day 4, an auxin minimum as visualized by low *DR5* expression (Figure 4, H–K) could be seen next to the cotyledon tip where embryogenic protrusions develop.

Auxin response reporters measure the sum of auxin signaling processes, and since BBM binds different types of auxin-pathway genes (Horstman et al., 2017b), we determined whether the enhanced *DR5* response observed in BBM overexpression lines can be explained by changes in IAA levels. WT seeds and seeds from two independent *35S:BBM-GR* lines differing in somatic embryo production rate were cultured with or without DEX for 3 d before measuring IAA and the IAA catabolite oxindole-3-acetic acid (oxIAA). Oxidation of IAA to oxIAA reduces auxin activity and plays an important role in maintaining auxin homeostasis (Stepanova and Alonso, 2016). Seedlings of both *35S:BBM-GR* lines treated with DEX showed higher IAA levels than the WT seedlings and *35S:BBM-GR* seedlings without DEX treatment (Figure 4L), but only the increase of IAA content in line 2 was significant compared to the WT control. The different IAA levels in these two lines might reflect the differences in penetrance of their somatic embryogenesis phenotypes (50% in line 1 and 100% in line 2).

The above data indicate that *BBM* overexpression induces a de novo auxin response on the adaxial cotyledon surface. The spatial localization of the *DR5* auxin response in DEX-treated *35S:BBM-GR* and WT seedlings started to diverge around the third day of culture, the time point at which *YUC3/YUC8* gene expression and IAA levels also increased in DEX-treated *35S:BBM-GR* cotyledons. This suggests that the enhanced auxin response observed in *35S:BBM-GR* seedlings is due, at least in part, to increased IAA biosynthesis. This increase in *YUC3/YU8* and *DR5* expression was followed a few days later by *DR5* and *YUC3/YUC8* expression minima at the site of multicellular somatic embryo formation on the cotyledon tip. Together this data suggest that enhanced/ectopic *YUC* expression and IAA biosynthesis coincides with the establishment of totipotent cell fate, but that multicellular somatic embryo development takes place in a low auxin response field.

### YUC3 and YUC8 are required for efficient BBM-mediated somatic embryogenesis

To determine the roles of *YUC3* and *YUC8* in BBM-induced somatic embryogenesis, we generated two independent *yuc3 yuc8* double mutant lines in a *35S:BBM-GR* background using CRISPR-Cas9 mutagenesis (Supplemental Figure S3). Both independent *yuc3 yuc8* mutants contained the same *yuc3^CR1^* mutation, an 848 bp deletion plus a 38 bp insertion that removed part of the promoter and first exon (Supplemental Figure S3, A and B). The *yuc8^CR1^* mutation has a 1 bp insertion downstream of and close to the translational start site, resulting in a premature stop codon (Supplemental Figure S3, A and B). The *yuc8^CR2^* mutant line has a 3 bp deletion at the same position as the *yuc8^CR1^* mutation resulting in loss of one amino acid (Supplemental Figure S3, A and B). This amino acid is not located in previously described functional domains (Supplemental Figure S3C) and might not affect the protein’s function. However, both the *yuc3^CR1^ yuc8^CR1^* and *yuc3^CR1^ yuc8^CR2^* mutants showed the reduced seed set phenotype that was previously described for the *yuc8-1* allele (Supplemental Figure S3D; Ståldal et al., 2012). This suggests that the single amino acid deletion in the *yuc8^CR2^* allele disrupts YUC8 function. Other than the reduced seed set phenotype, none of the two independent *yuc3^CR^ yuc8^CR^* double mutant lines showed obvious phenotypic differences from WT seedlings under standard growth conditions.

**Figure 5:**
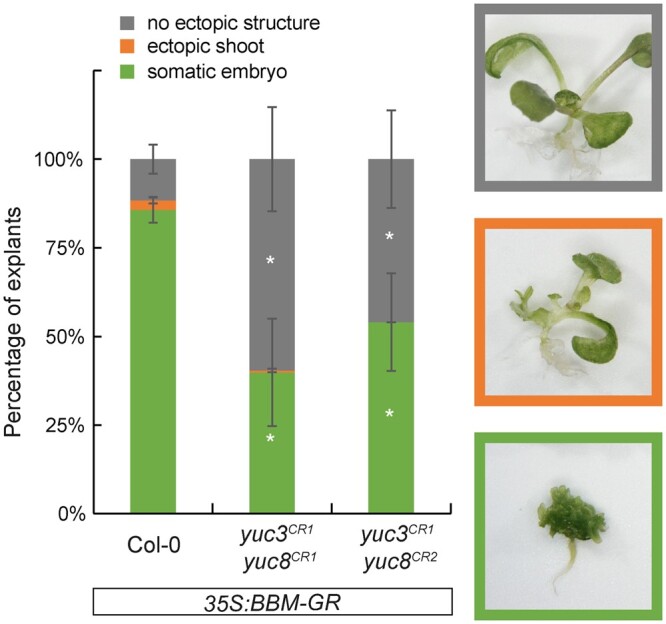
YUC-dependent auxin biosynthesis is required for efficient BBM-induced somatic embryogenesis. Regeneration phenotypes of 14-d-old explants from the indicated lines. The explants were categorized in three groups: explants with somatic embryos, explants with ectopic shoots and explants without any ectopic structures. Representative images are shown on the right. All seedlings were treated continuously with 10 μM DEX. Statistically significant differences in each category between the mutant lines and the *35S:BBM-GR* control line were determined using Student’s t test (*p* < 0.05) and indicated with asterisks. Error bars represent standard deviation of at least two biological replicates (*n* > 227).

To evaluate the effect of the *yuc3^CR^ yuc8^CR^* double mutants on BBM-induced somatic embryogenesis, we cultured control *35S:BBM-GR* seeds and seeds from the two *35S:BBM-GR yuc3^CR^ yuc8^CR^* lines for 14 d with 10 µM DEX and categorized the explants into three groups: explants with somatic embryos, explants with ectopic shoots but no somatic embryos, and explants without any ectopic structures (Figure 5). DEX-treated explants from both *35S:BBM-GR yuc3^CR^ yuc8^CR^* lines showed a statistically significant reduction in the capacity for somatic embryogenesis (ca. 50%) compared to the DEX-treated *35S:BBM-GR* control explants (ca. 90%). Ectopic shoot formation was not affected in the DEX-treated *35S:BBM-GR yuc3^CR^ yuc8^CR^* lines compared to the control. These results are in line with observations in 2,4-D-induced direct and indirect somatic embryo cultures, where mutation of different *YUC* genes was shown to be detrimental for somatic embryogenesis (Bai et al., 2013; Wójcikowska et al., 2013).

### Auxin biosynthesis is required in a narrow developmental window for efficient BBM-induced somatic embryogenesis

Auxin biosynthesis genes are direct targets of embryo identity transcription factors like BBM, LEC1, and LEC2 and these proteins also control each other’s expression through complex transcriptional feedback loops (Tian et al., 2020; Wójcik et al., 2020). Given the possibility that additional *YUC* genes might be directly or indirectly regulated during BBM-induced somatic embryogenesis, we used a pharmacological approach to inhibit overall YUC activity. This approach also allowed us to dissect the role of YUC-dependent IAA biosynthesis in time by performing time course inhibitor addition-removal experiments.

**Figure 6:**
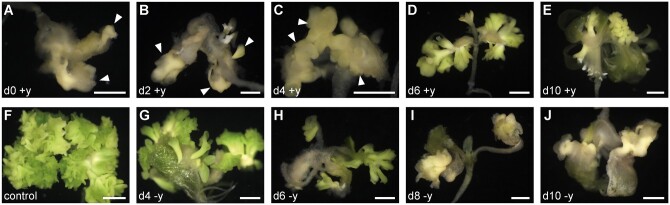
Auxin biosynthesis is required for BBM-mediated somatic embryogenesis. *35S:BBM-GR* seeds were grown for 14 d in the presence of DEX and imaged at the indicated time points. The YUC enzyme inhibitor yucasin (100 µM) was added or removed during the culture period as indicated (day +y or day −y). A–E, DEX-treated samples to which YUC inhibitor was added on Day 0, 2, 4, 6, 8, or 10. F, DEX-treated control sample. G–J, DEX-treated samples in which YUC inhibitor was added on Day 0 and then removed on Day 4, 6, 8, or 10. Scale bars, 1 mm.

*35S:BBM-GR* seeds were cultured for 14 d in liquid medium with 10 µM DEX to activate the BBM protein. The YUC enzyme inhibitor yucasin (Nishimura et al., 2014) or the more stable analog yucasin difluorinated analog (yucasin DF [YDF];100 µM; Tsugafune et al., 2017) were added to or removed from the cultures at different time points to determine when YUC-mediated IAA biosynthesis plays a role in BBM-induced somatic embryogenesis. After three to 4 d of culture, the cotyledon margins of DEX-treated *35S:BBM-GR* seedlings thicken due to increased cell division (Figure 1B). Multiple embryogenic protrusions develop from the adaxial surface of the cotyledon tip and margin around Day 6 of culture, followed by formation of histodifferentiated somatic embryos by 10 d of culture (Figures 1 and 6F). In contrast, the cotyledons of DEX-treated *35S:BBM-GR* seedlings treated with 100 µM YUC enzyme inhibitor from Days 0, 2, and 4 onward developed into white callus-like structures, with or without white, dense amorphous structures (Figure 6, A–C and F; Supplemental Figure S4, A–C and F). In contrast, seedlings from cultures treated with YUC enzyme inhibitor from Day 6 onward formed somatic embryos were similar to the control samples, except that the number of somatic embryos was greatly reduced compared to control cultures (Figure 6, D and E; Supplemental Figure S4, D and E). Continuous treatment of DEX in combination with lower YUC enzyme inhibitor concentrations also reduced somatic embryo formation in *35S:BBM-GR* seedlings, but to a lesser extent than with the 100 µM treatment (Supplemental Figure S5, A–E). The enhanced *DR5:GFP* expression in cotyledons of 4-d-old seedlings treated continuously with DEX was abolished after YUC enzyme inhibitor treatment (Supplemental Figure S5, K, L, and O), suggesting that YUC enzyme inhibitor treatment reduced BBM-induced IAA biosynthesis in the cotyledon.

Next, we performed YUC inhibitor removal experiments to more accurately define the time point at which inhibition of auxin biosynthesis affects the progression of somatic embryogenesis. DEX and YUC enzyme inhibitor were added on Day 0 of culture and then the inhibitor was removed on Days 4, 6, 8, or 10 of culture (Figure 6, G–J; Supplemental Figure S4, G–J). Somatic embryos developed on the cotyledons of DEX-treated *35S:BBM-GR* seedlings when YUC inhibitors were removed on or before Day 6, but the number of somatic embryos was reduced compared to nontreated control samples (Figure 6, G and H; Supplemental Figure S4, G and H). Somatic embryo formation could not be rescued when YUC enzyme inhibitor was removed after 6 d of treatment (Figure 6, I and J; Supplemental Figure S4, I and J).

Together these results suggest that YUC activity is essential for the normal progression of BBM-mediated somatic embryogenesis between the fourth and sixth day of culture. The YUC inhibitor concentrations that affect somatic embryo formation (25–100 µM; Supplemental Figure S5, A–E) are higher than those that affect root development in WT plants (1–10 µM; He et al., 2011), but similar to the concentration range (20–100 µM) that complemented the *YUC1* overexpression phenotype (Nishimura et al., 2014). This suggests that BBM induces relatively high IAA levels in cotyledons or that cotyledons and developing somatic embryos are less sensitive to YUC enzyme inhibition than other tissues.

TAA/TAR proteins convert TRP to IPyA, which is then converted to IAA by YUC proteins. The *TAA1* gene is also bound by BBM during BBM- and 2,4-D-induced somatic embryogenesis but was not transcriptionally-regulated by BBM during the first 2 d of culture (Figure 2, C and D). However, blocking TAA1/TAR enzyme activity in *35S:BBM-GR* seedlings with kynurenine (kyn), a chemical inhibitor of TAA1/TAR activity (He et al., 2011) severely impaired somatic embryo formation (Supplemental Figure S5, F–J) and also abolished the BBM-induced *DR5* response (Supplemental Figure S5, M–O). This inhibitory effect was not observed when kyn was added to the medium on Day 6 of culture (Supplemental Figure S6, D and E) or when kyn was removed by Day 8 of culture (Supplemental Figure S6, F–I), although fewer embryos developed than in the control samples. Thus *TAA1/TAR*-mediated auxin biosynthesis is also required for BBM-induced somatic embryogenesis, although the window in which TAA1/TAR enzymes are required is slightly broader than for YUC enzymes.

### Auxin biosynthesis is required for the maintenance of BBM-induced totipotency

To determine how reduced IAA levels affect the progression of BBM-mediated somatic embryogenesis, we examined the development of auxin inhibitor-treated explants using thin sections and embryo identity reporters.

**Figure 7:**
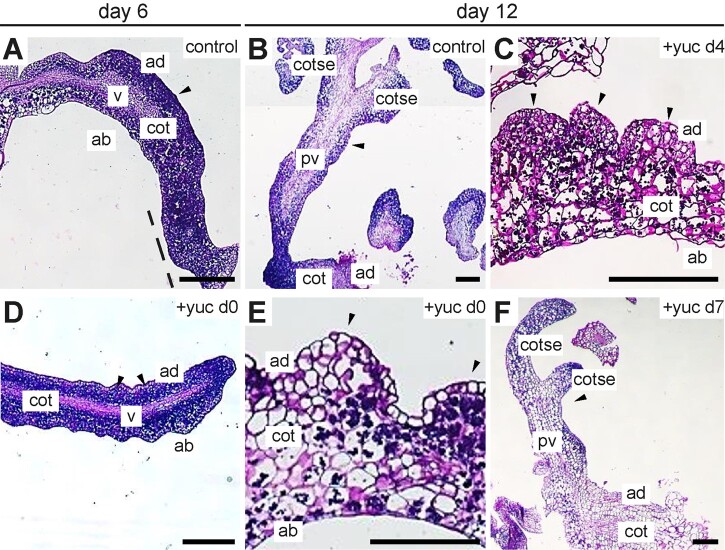
YUC-dependent auxin biosynthesis is required for the formation of histodifferentiated somatic embryos. Light micrographs of thin cross sections of the cotyledons of DEX and YUC inhibitor (yucasin)-treated *35S:BBM-GR* explants fixed on the days indicated above the images. The day of culture and the yucasin treatment (100 µM) is shown above and in the image panels, respectively. A and B, Explants from control samples treated with DEX from Day 0 until the end of the culture on Day 14. Panel B is a composite of different images from the same section. C–F, Explants from samples treated with DEX from Day 0 to Day 14, to which YUC enzyme inhibitor was added on Day 0 (D and E), Day 4 (C), or Day 7 (F). Black arrowhead, growth protrusions (A, C–E) and somatic embryos (B and F); cot, cotyledon; cotse, cotyledons of somatic embryos; v, vascular (A and D); pv, provascular tissue (B and F); dotted line, proliferating cotyledon tip. Scale bars, 200 μm.

*35S:BBM-GR* seeds were germinated in medium containing DEX (control) with or without YUC enzyme inhibitor, which was added to the cultures during (Days 0 and 4) or after (Day 7) the critical time point for somatic embryo development. Thin sections were made six and 12 d after the start of culture. Thin sections of DEX-treated seedling cotyledons showed that the mesophyll and vascular cells had divided prolifically during the first 6 d of culture (Figure 7A). The proliferating adaxial mesophyll cells and cotyledon tip formed a continuous mass of cytoplasm-rich cells, which are characteristic for totipotent/meristematic cells (Huang and Yeoman, 1984; Prime et al., 2000; Kurczyńska et al., 2007; Verdeil et al., 2007; Godel-Jedrychowska et al., 2020). Callus-like cells, characterized by their reduced cytoplasmic staining, were visible on the adaxial surface of the cotyledon in the same explants (Figure 7A). By Day 12 of culture, the DEX-treated seedlings had formed (secondary) somatic embryos with defined apical–basal polarity (Figure 7B). When YUC enzyme inhibitor was added with DEX at the start of culture, the seedlings still produced cytoplasm-rich cells on the cotyledon surface, but with less overall cell proliferation compared to DEX-treated samples (Figure 7D). In addition, interspaced cell clusters formed along the adaxial surface of the cotyledon instead of the continuous band of proliferating cells observed in DEX-treated seedlings. These cell clusters became more callus-like by the 12th day of culture (Figure 7E). The cells in these callus-like clusters were covered by loosely connected epidermal cells, rather than densely packed cells in the control samples, indicating that they lost their capacity for meristematic/totipotent cell proliferation. The cotyledons of seedlings treated with YUC inhibitor on Day 4 resembled cotyledons from seedlings treated with inhibitor from Day 0 onward (Figure 7C). When YUC inhibitor was added on Day 7 of culture, somatic embryos with visible apical–basal polarity were formed on the cotyledons (Figure 7F), but the number of somatic embryos was reduced compared to the DEX-treated control. These data indicate that auxin biosynthesis is not absolutely required for the de novo induction of meristematic/totipotent cell proliferation, but rather is required to sustain these meristematic/totipotent cell divisions. These results also support the idea that auxin biosynthesis is also required after Day 6 of culture for efficient differentiated somatic embryo formation.

**Figure 8:**
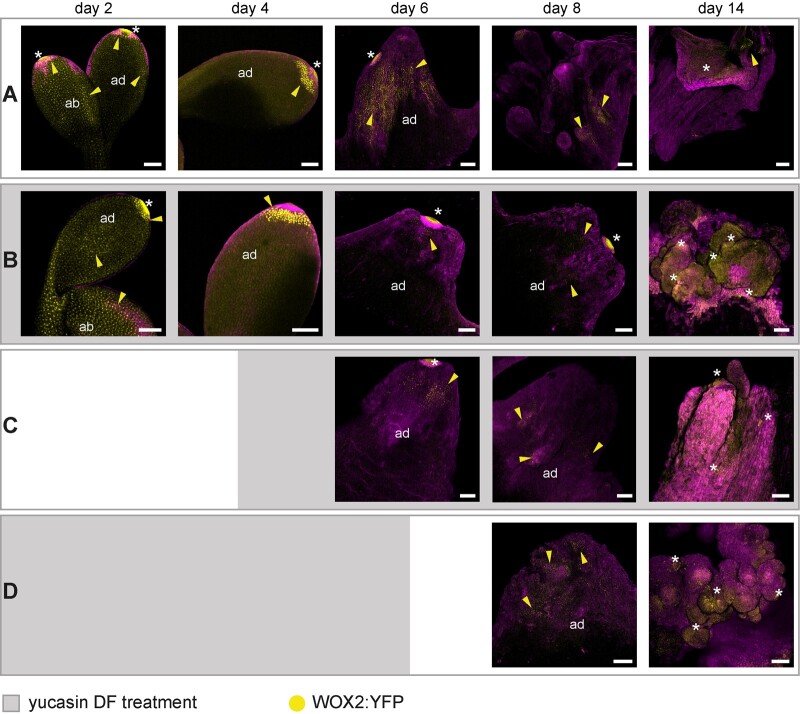
Auxin biosynthesis is required to maintain *BBM*-induced totipotency. Confocal laser scanning micrographs of cotyledon/cotyledon tips of *35S:BBM-GR* explants grown with DEX, with or without the YUC enzyme inhibitor YDF (100 µM). A, Control DEX-treated explants. B–D, Explants treated with DEX and YDF, which was added or removed on the days indicated by gray blocks in each row. Samples were counter stained with SR2200 (magenta). The day of culture is indicated in the panels. Yellow arrowheads, *WOX2* expression (yellow signal). Asterisks, autofluorescence. Scale bars, 100 μm.

To determine how reduced IAA levels alter embryo fate during BBM-induced somatic embryogenesis, we followed the expression of the *WOX2:NLS-3xYFP* embryo identity reporter in DEX-treated *35S:BBM-GR* seedlings that were cultured in the presence of absence of YUC enzyme inhibitors. WOX2-YFP expression in seedlings treated continuously from Day 0 with 100 µM YUC enzyme inhibitor was similar to that of the control seedlings until Day 4 of culture (Figures 1F and 8B). The number of WOX2-YFP-positive seedlings decreased to half that of the control by Day 8 of culture and to zero by Day 14 (Supplemental Table S1; Figure 8B). When YDF was added on Day 4 of culture, the initial proportion of WOX2-YFP-expressing seedlings on Days 6 and 8 was similar to that of the DEX-treated control, but then decreased to zero on Day 14 (Supplemental Table S1; Figure 8C). Likewise, when YDF was added on Day 0 and then removed on Day 6 of culture, the number of seedlings initially showing WOX2-YFP expression was similar to the control, but then decreased to zero by Day 14 of culture (Supplemental Table S1; Figure 8D).

Taken together, these histology and cell fate experiments confirmed our observations on whole mount samples that is, that YUC-dependent IAA biosynthesis is not required for the initiation of embryo identity at the cotyledon tip in BBM overexpression lines, but is required later, in a narrow developmental window between Days 4 and 6 of culture, to maintain embryo identity and promote the development of embryogenic cell protrusions into histodifferentiated embryos. In the absence of YUC activity these embryogenic cells develop into callus-like structures.

## Discussion

Ectopic expression of the AIL transcription factor BBM induces spontaneous adventitious organ formation (pluripotency) and embryogenesis (totipotency; Gordon-Kamm et al., 2019; Vijverberg et al., 2019). In WT plants, in vitro adventitious organ formation and somatic embryogenesis usually rely on exogenous auxin application, either alone or in combination with other hormones or abiotic stress treatments. A genetic relationship between BBM-like AILs and auxin in shoot and root meristem development, as well as binding and/or direct transcriptional regulation of *YUC* genes by AIL-family members has been shown (Pinon et al., 2013; Santuari et al., 2016), but neither has been described in the context of induced pluripotent or totipotent growth. Here we show that BBM regulates *YUC* gene expression and that YUC-dependent auxin biosynthesis has essential, but relatively late functions in BBM-mediated somatic embryogenesis. Our data suggest a two-step model in which BBM-induces expression of embryo identity genes like *LEC1, LEC2*, and *FUSCA3* (*FUS3*) to establish cell totipotency (Horstman et al., 2017b), followed by induction of auxin biosynthesis to maintain embryo division and growth.

### Multiple roles for auxin biosynthesis

Here we show that ectopic BBM expression induces expression of the canonical auxin biosynthesis pathway genes *YUC3* and *YUC8* (Figure 2D). Both of these genes are direct BBM targets in 2,4-D and BBM-induced somatic embryo cultures (Figure 2, A and B). *BBM* is expressed in the seedling root tip and throughout the zygotic embryo as early as the four-cell stage and becomes basally localized from the heart stage onward (Galinha et al., 2007; Horstman et al., 2015). Both *YUC3* and *YUC8* are expressed in the seedling root tip (Chen et al., 2014; Santuari et al., 2016; Figure 3), and in the zygotic embryo *YUC3* is expressed in the suspensor and *YUC8* in the basal region of the embryo proper (Robert et al., 2013). This overlap in *BBM* and *YUC3*/*YUC8* expression suggests that BBM also regulates *YUC3* and *YUC8* expression during zygotic embryogenesis and root development in planta.

Reporter analysis showed that *35S:BBM-GR* overexpression induces *YUC3* and *YUC8* expression in the root and hypocotyl, followed by expression in the cotyledons (Figure 3). The expansion of BBM-induced ectopic *YUC3*/*YUC8* reporter expression from the below ground to the above-ground organs reflects the gradual increase in transcript levels detected by qPCR (Figure 2D). The increase in *YUC* expression in roots and cotyledons was also mirrored by increased *DR5* expression in the same organs and by increased IAA biosynthesis (Figure 4). Together these results suggest that BBM induces enhanced and ectopic auxin biosynthesis gene expression and a concomitant increase in auxin levels.

We also observed that embryogenic protrusions develop in areas of low (*DR5*) auxin response (Figure 4) and low *YUC3*/*YUC8* expression (Figure 3). In Arabidopsis, *DR5* expression and auxin accumulation (as measured by the *R2D2* [Ratiometric version of 2D2’s] reporter; Liao et al., 2015) are only reliably detected starting at the eight-cell embryo stage. This initial auxin response in the embryo proper is largely due to PIN-mediated auxin transport from the suspensor and from the surrounding maternal tissues (Friml et al., 2003; Robert et al., 2013). *YUC* and *TAA1/TAR* auxin biosynthesis genes are expressed later in zygotic embryos, in the embryo proper and suspensor from the 16-cell embryo stage onward (Stepanova et al., 2008; Robert et al., 2013). In *35S:BBM-GR* explants, *DR5* and *YUC3* are initially expressed throughout the cotyledon and *YUC8* in the cotyledon vasculature. Later, *DR5* and *YUC3*/*YUC8* expression is absent at the sites where *WOX2-YFP* expression is ectopically induced and where multicellular embryos emerge on the cotyledon tip and margin (Godel-Jedrychowska et al., 2020; Figures 1, F–I, 3, J and T, and 4, H–K). Reduced *DR5* and *YUC* expression might simply reflect a switch in development from single or few-celled embryogenic structures to larger embryogenic clusters, analogous to early preglobular stage zygotic embryos, where neither *DR5* nor characterized *YUC* genes are expressed. Alternatively, we have shown previously that this decrease in *DR5* expression is accompanied by and requires increased callose production in plasmodesmata adjacent to sites of *WOX2-YFP* expression (Godel-Jedrychowska et al., 2020). Blocking callose production in DEX-treated *35S:BBM-GR* seedlings prevents the formation of an auxin response minimum and completely blocks somatic embryo development. We hypothesized that auxin accumulation must be reduced locally to allow organized embryo growth and that callose deposition in surrounding plasmodesmata prevents passive auxin re-entry into these cells. Thus, a combined action of reduced auxin accumulation, reduced local auxin biosynthesis and reduction of the size of molecules that can pass through plasmodesmata might create a low auxin field that promotes the growth of multicellular embryogenic growth protrusions. Auxin biosynthesis inhibitor experiments showed that auxin is required later for further growth of these embryogenic protrusions into differentiated embryos; blocking YUC-dependent auxin biosynthesis results in conversion of embryogenic cells to callus-like structures rather than somatic embryos. At this point, callose deposition and *WOX2-YFP* expression colocalize in the same cells, as embryogenic protrusions increase in size and differentiate into somatic embryos (Godel-Jedrychowska et al., 2020). Together these observations suggest a two-step dynamic and local regulation of auxin to allow 1) development of multicellular embryogenic cell clusters in a low auxin/auxin response area, followed by 2) development of these structures into histodifferentiated embryos with zygotic embryo-like auxin responses.

### A positive transcriptional feedback loop for somatic embryogenesis

Somatic embryo formation was completely abolished when DEX-treated *35S:BBM-GR* explants were treated continuously or before the sixth day of culture with YUC enzyme inhibitors, but the somatic embryogenesis rate in the *35S:BBM-GR yuc3^CR^ yuc8^CR^* lines was only reduced to about half of the control *35S:BBM-GR* line (Figure 5). This result suggests that YUC3 and YUC8 are not the only YUC enzymes required for BBM-induced somatic embryogenesis. Previously we found that BBM also binds the *LEC1*, *LEC2*, and *FUS3* transcription factor genes (Horstman et al., 2017b). Ectopic *LEC1* expression was also induced in DEX-treated *35S:BBM-GR* seedlings during the first day of culture. LEC1 and LEC2 expression in seedlings induces, respectively, *YUC8* and *YUC10* (Junker et al., 2012; Huang et al., 2015) and *YUC1*, *YUC4*, and *YUC10* expression (Wójcikowska et al., 2013). LEC2 and FUS3 also cooperatively promote *YUC4* expression during lateral root formation (Wójcikowska et al., 2013; Tang et al., 2017). The LEC transcription factors might partly compensate for the reduced auxin biosynthesis in *yuc3^CR^ yuc8^CR^* mutant lines by inducing expression of other *YUC* genes. The known positive transcriptional interactions between the BBM and LEC transcription factors and their respective target genes (Horstman et al., 2017a; Tian et al., 2020) suggest that a positive feedback loop is established that maintains both embryo identity and auxin biosynthesis during BBM-induced somatic embryogenesis.

### Auxin requirement during embryogenesis

In Arabidopsis, *YUC* gene expression is activated during 2,4-D-induced somatic embryogenesis in explants undergoing direct and indirect somatic embryogenesis (Bai et al., 2013; Wójcikowska et al., 2013). During 2,4-D-induced direct somatic embryogenesis from Arabidopsis immature zygotic embryo explants, overexpression of *LEC2* can compensate for treatment with a suboptimal 2,4-D concentration or for treatment with auxins that are poor inducers of somatic embryogenesis, like IAA or 1-naphthaleneacetic acid (Wójcikowska et al., 2013). The *lec1* and *lec2* loss-of-function mutants show a severe reduction of the number of embryogenic explants in the presence of 2,4-D, as well as a shift from direct to indirect (callus-derived) somatic embryogenesis (Gaj et al., 2005). Conversely, ectopic expression of *LEC2* in the presence of an optimal concentration of 2,4-D negatively affects somatic embryo formation, as it delays and reduces embryo induction and induces callus and shoot-like structures instead of somatic embryos (Ledwoń and Gaj, 2009). Although IAA levels were not measured directly in these studies, these results suggest that tight regulation of auxin levels is required to promote somatic embryogenesis: both too little and too much endogenous or exogenous auxin can inhibit somatic embryo formation, absolutely and/or in favor of shoot or callus production (Ledwoń and Gaj, 2009).

The above studies on 2,4-D-induced somatic embryogenesis in WT and different LEC backgrounds demonstrate a role for YUC-dependent auxin biosynthesis in promoting efficient somatic embryogenesis. However, these studies did not determine when and for which aspect of somatic embryogenesis YUC-dependent IAA biosynthesis was required. Our analyses indicated that both *YUC* expression and IAA levels increase as early as 3 d after BBM activation (Figures 3 and 4L). These changes also correspond with onset of embryo marker gene expression, including *WOX2-YFP* (Figure 1). However, our pharmacological experiments using YUC enzyme inhibitors showed that YUC-TAA1/TAR-dependent IAA biosynthesis is not required at this time point for the re-initiation of totipotent growth (Figure 6). YUC-dependent IAA biosynthesis is required later, between Days 4 and 6 of culture, for the maintenance of embryo identity and for embryo growth and histodifferentiation. In explants treated continuously or up until the sixth day of culture with YUC enzyme inhibitors, cytoplasm-rich embryogenic protrusions do not progress to patterned embryos, but rather form callus-like structures (Figure 7). How does BBM-induced auxin biosynthesis maintain embryo growth and development? Recently, Karami et al. (2021a) showed that induction of cell totipotency during 2,4-D and *35S:AHL15*-induced somatic embryogenesis does not require the auxin efflux and influx machinery. Rather, auxin transport is required later, for the proper transition of embryogenic cells to multicellular embryos and for correct embryo differentiation. Similarly, it is likely that endogenous auxin supplied by BBM signaling is also required to establish the auxin gradients needed for embryo outgrowth and patterning.

During zygotic embryogenesis, YUC and TAA1 genes are expressed relatively late, during the transition from the globular/heart stage to the torpedo stage, where they are required for correct embryo patterning (Stepanova et al., 2008; Robert et al., 2013). TAA1/TAR and YUC genes are expressed earlier in the surrounding maternal ovule and seed coat, but maternally supplied auxin only appears to be required for proper embryo patterning (Robert et al., 2018). Although a complete description of all *YUC* genes and other TRP-independent IAA synthesis genes during zygotic embryogenesis is currently not available, this data, together with our observations on BBM-induced totipotency suggest that YUC-dependent auxin biosynthesis is not required for the initiation of embryo identity per se. In contrast, TRP-independent IAA biosynthesis has been shown to be essential for early zygotic embryo viability and patterning (Wang et al., 2015). TRP-independent auxin biosynthesis genes have not been identified as direct BBM targets, but might act downstream of other BBM target genes. Recently, Li et al. (2021) described a developmental pathway in which MATERNAL EFFECT EMBRYO ARREST45 induces the *AIL* gene *AINTEGUMENTA*, which in turn regulates *YUC* expression in the ovule integument to control embryo size. These results are in line with our observations on the role of YUC-dependent auxin biosynthesis in maintaining embryogenic cell divisions in vitro and suggest that similar seed functions might be co-opted by embryo identity transcription factors like BBM in embryogenic explants.

## Conclusion

The importance of auxin for in vitro somatic embryogenesis is apparent in its widespread use as an exogenous inducer and in the requirement for endogenous auxin for efficient somatic embryo production. “Totipotency” transcription factors are not only rapidly induced in response to 2,4-D, but also induce somatic embryogenesis in the absence of exogenous auxin (Ledwoń and Gaj, 2009, 2011; Horstman et al., 2017a; Tian et al., 2020). These transcription factors also bind to and/or transcriptionally regulate auxin biosynthesis genes, making them good candidates for direct regulators of auxin biosynthesis in different somatic embryogenesis systems. We show that YUC-dependent auxin biosynthesis is required to maintain somatic embryo identity and promote growth, but not for the cell fate transition to embryogenesis. De novo induction of both embryo identity transcription factors and auxin biosynthesis therefore ensures that embryogenic cells proliferate and develop into somatic embryos.

## Materials and methods

### Plant material and growth conditions

The *35S:BBM-GR*, *WOX8gΔ:NLS-venusYFP3* (referred to here as *WOX2:NLS-3xYFP*), *YUC8:GUS, YUC3:GUS, YUC3:erGFP*, *DR5v2:ntdTomato*, *DR5:GUS*, and *DR5:GFP* lines were described previously (Benková et al., 2003; Růžička et al., 2007; Breuninger et al., 2008; Passarinho et al., 2008; Chen et al., 2014; Liao et al., 2015; Santuari et al., 2016). Due to *BBM* silencing upon outcrossing (Horstman et al., 2017b), the majority of *35S:BBM-GR* lines containing reporter constructs were made by either transforming the *35S:BBM-GR* vector to the reporter line (*YUC8:GUS* and *WOX2:NLS-3xYFP*) and then selecting highly embryogenic lines, or transforming the reporter vectors (*DR5v2, YUC3:erGFP*) to an existing embryogenic *35S:BBM-GR* line. In the latter case, the transgenic lines were selected based on reporter expression. For the *DR5:GUS* and *DR5:GFP* reporter lines, crosses were made with a homozygous *35S:BBM-GR* line and the progeny selected over four generations until nonsilenced homozygous lines with at least 90% penetrance of embryogenic explants and 100% reporter gene expression were recovered.

Seeds were sterilized with liquid bleach as described previously (Horstman et al., 2017a, 2017b). For liquid cultures, sterilized seeds were dispensed in 190 mL containers (Greiner, Kremsmünster, Austria) with 30 mL liquid half-strength Murashige and Skoog (1/2 MS-10) medium (Murashige and Skoog, 1962) with 1× MS vitamins, pH 5.8, and 1% sucrose (w/v). The liquid cultures were stratified at 4°C in the dark for up to 48 h before transfer to a rotary shaker (60 rpm) at 25°C (16 h light/8 h dark cycle) for the indicated time. For solid medium cultures, sterilized seeds were cultured at 21°C (16-h light/8-h dark cycle) on 1/2 MS-10 medium with 0.8% (w/v) agar.

### Chemical treatments

DEX (Sigma, St. Louis, MO, USA) was dissolved in 70% ethanol and used at a final concentration of 10 µM in all experiments. Yucasin (Sigma; Nishimura et al., 2014), YDF (Tsugafune et al., 2017; provided by Hayashi lab) and kyn (Sigma) were all dissolved in DMSO and were added to the solid and liquid culture medium as described in the text. Mock-treated samples contained the same volume of ethanol or DMSO. The liquid medium and chemicals were refreshed every 6–7 d. Analysis of somatic embryogenesis phenotypes was performed with >3 replicates with >100 explants per treatment. The phenotypes shown were observed in 100% of the explants.

### CRISPR-Cas9 mutagenesis

To avoid *BBM* silencing upon outcrossing (Horstman et al., 2017b), *yuc3 yuc8* double mutants were generated by CRISPR-Cas9 mutagenesis directly in the *35S:BBM-GR* background rather than by crossing with T-DNA mutants. CRISPR-Cas9 mutagenesis of *YUC3* and *YUC8* was performed using the *U6-26* promoter for the single-guide RNAs (sgRNAs), an *RPS5A* promoter-driven Arabidopsis codon-optimized *Cas9* gene (Fauser et al., 2014), and FAST-Red selection (Castel et al., 2019), all in vector *pICSL4723* (Weber et al., 2011; Wang et al., 2019)*.* Two sgRNAs targeting *YUC3* and two sgRNAs targeting *YUC8* were assembled into one vector to obtain *yuc3^CR^ yuc8^CR^* double mutant lines. The sgRNAs and mutant genotyping primers are listed in Supplemental Table S2. The CRISPR-Cas9 vectors were transformed to a highly embryogenic *35S:BBM-GR* line. Two double *yuc3 yuc8* mutant lines, each with the same *yuc3* mutation and a different *yuc8* mutation were obtained (Supplemental Figure S3). Homozygous T4 CAS9-free *yuc3* and *yuc8* mutants were used for the analysis. Analysis of somatic embryogenesis efficiency was performed with at least two technical replicates with >99 explants per mutant line.

### Transformation

All constructs were transformed using the floral dip method (Clough and Bent, 1998). Transgenic T1 seeds from CRISPR transformants were selected based on FAST-Red expression (Castel et al., 2019). Transgenic T1 seedlings with reporter lines were selected as described above. Homozygous mutant lines were used in all analyses.

### Quantitative real-time RT-PCR

RNA was isolated using the InviTrap Spin plant RNA mini kit (Invitek Molecular, Washington, DC, USA; # 1064100300) with the addition of 25 µL Plant RNA isolation Aid (Ambion, Austin, TX, USA), followed by a DNAse treatment (TURBO DNA-free kit; Invitrogen, Waltham, MA, USA). cDNA was synthesized using the iScript cDNA synthesis kit (Bio-Rad, Hercules, CA, USA) following the manufacturer’s instructions. Quantitative real-time RT-PCR (RT-qPCR) was performed using a BioRad MyiQ PCR machine with the SYBR green mix as described in Horstman et al. (2015). Relative gene expression was calculated with the 2^−ΔΔCT^ method (Livak and Schmittgen, 2001) using the nonDEX treated (mock) samples as calibrators and the *SAND* gene (Czechowski et al., 2005) as the reference. Three biological replicates comprising germinating seeds/seedlings were used for each treatment. Statistically significant changes in gene expression levels were determined using Student’s t test *P* < 0.05. The qPCR DNA primers are shown in Supplemental Table S3.

### Histology

Fresh material for sectioning was fixed overnight at 4°C in 3:1 absolute ethanol:glacial acetic acid and then dehydrated stepwise from 70% to 100% ethanol. The fixed material was infiltrated in Steedman’s wax and then sectioned and stained with 0.05% Toluidine Blue (w/v) as previously described (Wrobel et al., 2011). Images were taken with a Nikon Eclipse Ni microscope with a Nikon DS-Fi1 camera and NIS Elements L software (Nikon). About 9–12 explants per treatment were observed.

### Microscopy

Confocal laser scanning microscopy was performed as previously described (Soriano et al., 2014; Horstman et al., 2017b). Samples were fixed with 4% (w/v) para-formaldehyde, counterstained with 0.1% (v/v) SCRI Renaissance 2200 (SR2200; Musielak et al., 2016) and then stored at 4°C for up to 2 weeks before imaging. Fluorescence was observed using a Leica SPE DM5500 confocal microscope using the LAS AF software. SR2200 was exited with the 405-nm laser line and fluorescence emission detected between 415 and 476 nm. GFP was excited with the 488-nm laser line and light emission detected between 505 and 540 nm. YFP was excited with the 488-nm laser line and detected between 517 and 597 nm. tdTomato was excited with the 561-nm laser line and light emission detected between 571 and 630 nm. Brightness/contrast adjustment was done in the LAS AF software and image cropping was done in ImageJ. About 9–20 explants were analyzed for each treatment. The images represent the majority of the examined explants or as noted in Supplemental Table S1.

GUS assays were performed for up to 22 h at 37°C, as previously described (Sieburth and Meyerowitz, 1997) using 2.5 mM potassium ferri and ferrocyanide. GUS-stained tissues were cleared in HCG (water:chloral hydrate:glycerol, 25:55.7:8.3; w/w) and then observed using a Nikon Optiphot microscope with differential interference contrast optics. Images were recorded with a Nikon DS-Fi1 camera and processed using NIS-Elements D 3.2 software and ImageJ. Light microscopy was performed using a ZEISS Stemi SV 11 microscope. The GUS assay was repeated 2 times with at least 40 explants examined for each timepoint. The images represent the majority of the examined explants.

### IAA measurements

Seeds from WT Col-0 and two independent *35S:BBM-GR* lines (two replicates per line) were grown for 24 h in liquid 1/2 MS-10 medium and then grown for an additional 3 d in the presence or absence of 10 µM DEX. IAA extraction and measurements were performed as in Ruyter-Spira et al. (2011) using ca. 100–250 mg fresh weight per sample.

## Accession numbers

The previously published chromatin immunoprecipitation sequencing (ChIP-seq) data and data analysis (Horstman et al., 2015) can be downloaded from the Gene Expression Omnibus (GEO; GSE52400).

## Supplemental data 

The following materials are available in the online version of this article.

**Supplemental Figure S1.** Confocal images of control *35S:BBM-GR WOX2:YFP* explants.

**Supplemental Figure S2.** The BBM DNA binding motif in the *YUC3* promoter is required for *YUC3* expression in root meristems and BBM-induced *YUC3* ectopic expression.

**Supplemental Figure S3.** CRISPR-Cas9-induced *yuc3* and *yuc8* alleles.

**Supplemental Figure S4.** Magnified images of 14-d-old DEX and DEX+YUC inhibitor-treated *35S:BBM-GR* explants.

**Supplemental Figure S5.** Auxin biosynthesis inhibitors block somatic embryo formation and auxin response.

**Supplemental Figure S6.** TAA1/TAR auxin biosynthesis is required for BBM-mediated somatic embryogenesis.

**Supplemental Table S1.** Percentage of *35S:BBM-GR WOX2:NLS-3xYFP* seedlings with YFP signal in the cotyledon tip or growth protrusion.

**Supplemental Table S2.** Single-guide RNAs used for CRISPR-Cas9 mutagenesis and primers used for genotyping CRISPR mutants.

**Supplemental Table S3.** DNA primers used for RT-qPCR.

**Supplemental Data **Set S1.**** BBM direct target genes as determine by ChIP-seq.

## Supplementary Material

kiab558_Supplementary_Data

## Notes

M.L., J.W.-M., I.H., A.H., B.C., and R.R. performed experiments and analyzed the data. M.L., J.W.-M., I.H., A.H., B.C., R.R., G.C.A., and K.B. contributed to the experimental set-up and data interpretation. M.L. and K.B. wrote the manuscript with input from all authors. K.B. agrees to serve as the author responsible for contact and ensures communication.

The author responsible for distribution of materials integral to the findings presented in this article in accordance with the policy described in the Instructions for Authors (https://academic.oup.com/plphys/pages/general-instructions) is: Kim Boutilier (kim.boutilier@wur.nl).
