# Supplementary material for: Auxin biosynthesis maintains embryo identity and growth during BABY BOOM-induced somatic embryogenesis
Source: Plant Physiol. 2021 Dec 1;188(2):1095–110. doi: 10.1093/plphys/kiab558 (PMC8825264; doi:10.1093/plphys/kiab558)
Supplement: kiab558_Supplementary_Data [file kiab558_supplementary_data.zip › PP2021RA01097R1_Supplemental_Figures and Tables.pdf]

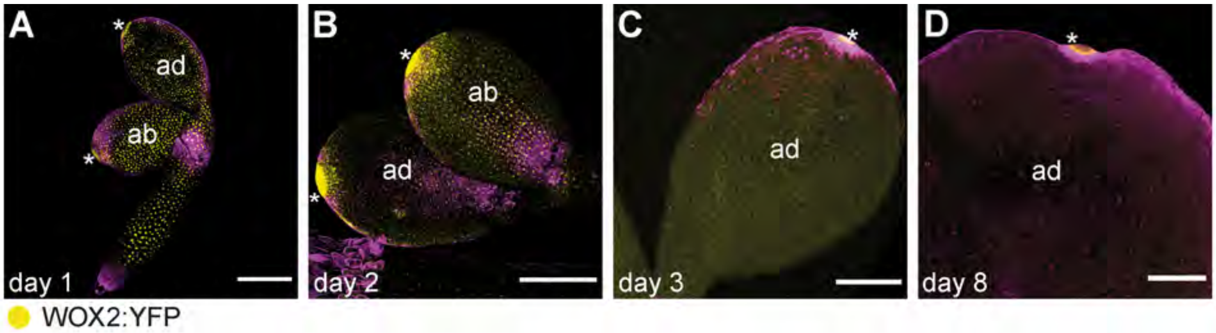

Supplemental Figure S1. Confocal images of control *35S:BBM-GR WOX2:YFP* explants. A-D. Confocal laser scanning micrographs of *WOX2* expression in an imbibed embryo (A) and cotyledons (B-D) on the indicated day of culture. The image in (D) is from a non-embryogenic explant. Samples were counter stained with SR2200 (magenta signal). *WOX2* expression (yellow signal). The day of culture is indicated in the panels. Asterisks, autofluorescence. ab, abaxial side; ad, adaxial side. Scale bars, 100 µm.

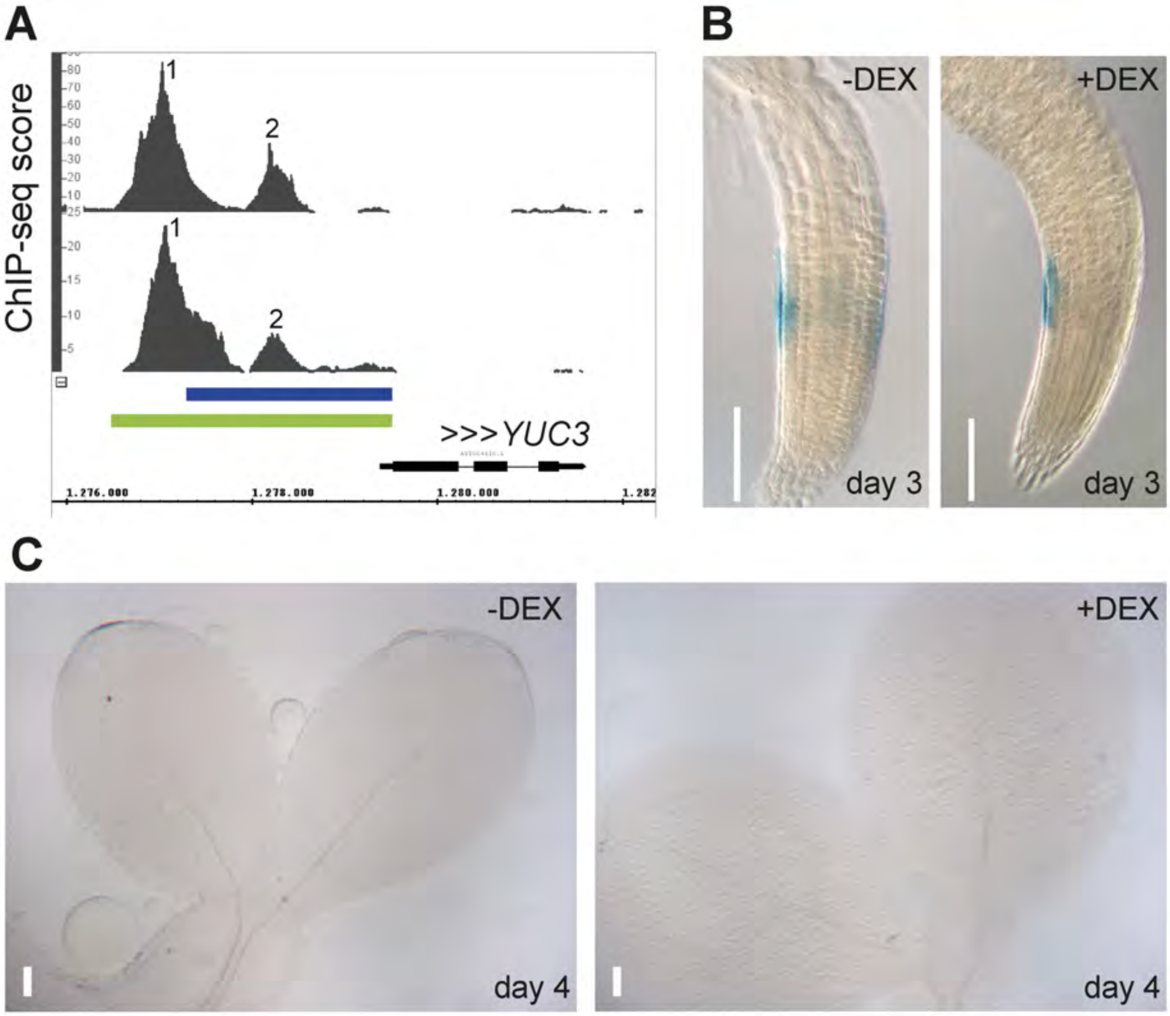

Supplemental Figure S2. The The BABY BOOM (BBM) DNA binding motif in the *YUC3* promoter is required for *YUC3* expression in root meristems and BBM-induced *YUC3* ectopic expression. A. BBM chromatin immunoprecipitation sequencing (ChIP-seq) profiles for *YUC3*, as shown in Figure 2. The promoter sizes of the *YUC3* reporters are indicated by the coloured bars. Blue line, *YUC3:GUS*; Green line, *YUC3:erGFP*. The consensus BBM binding motif is located in the middle of peak 1. B. GUS expression in roots from three-day-old *35S:BBM-GR YUC3:GUS* seedlings treated without dexamethasone (-DEX) and with DEX (+DEX). C. GUS expression in cotyledons from four-day-old *35S:BBM-GR YUC3:GUS* seedlings treated without (-DEX) and with DEX (+DEX). Scale bars, 100 μm.

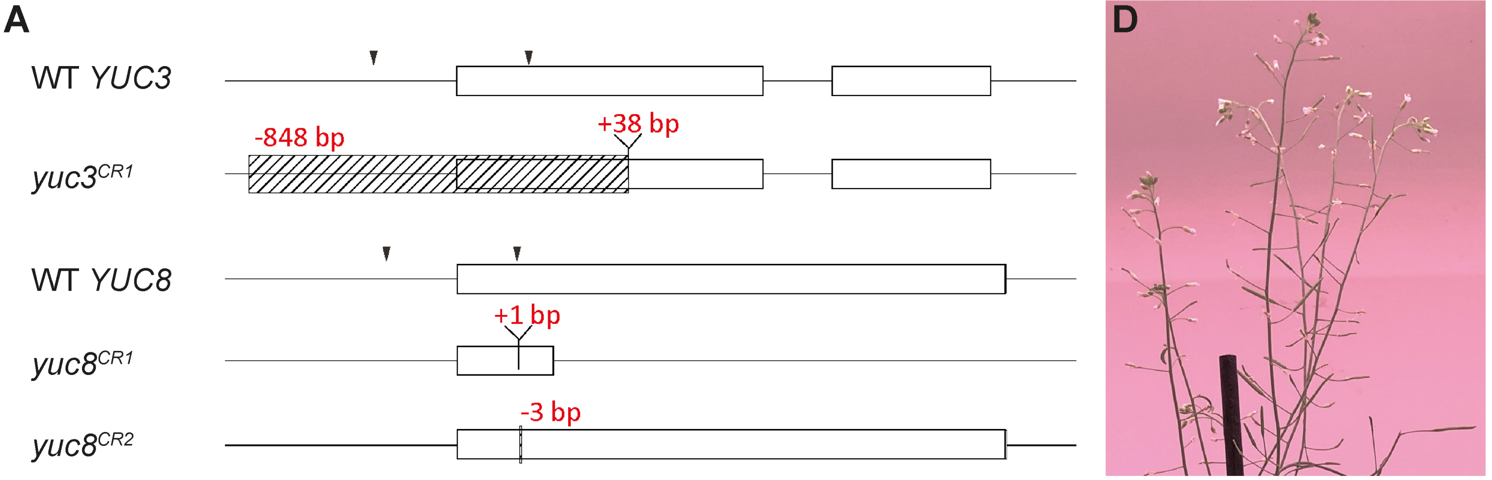

B

Allele	Sequences
yuc3 CR1 yuc8 CR1	
WT *YUC3*	cattttggtagttct.......cca atttgcatcaagtcgtcttc ........GCTGCGGGCTTGAAACGTGA AGG...........CAGTCCGCTAAATATG
yuc3 CR1	cattttg- -848 bp deletion + 38 bp insertion -AAATATG
WT *YUC8*	gaaaaatattcaacgccaca tgg ....CCTTTCG - TTGTTCTCGAGAGAGC
yuc8 CR1	gaaaaatattcaacgccaca tgg ....CCTTTCGGTTGTTCTCGAGAGAGC
yuc3 CR1 yuc8 CR2	
WT *YUC3*	cattttggtagttct.......cca atttgcatcaagtcgtcttc ........GCTGCGGGCTTGAAACGTGA AGG...........CAGTCCGCTAAATATG
yuc3 CR1	cattttg- -848 bp deletion + 38 bp insertion -AAATATG
WT *YUC8*	gaaaaatattcaacgccaca tgg ....CCTTTCGTTGTTCTCGAGAGAGC
yuc8 CR2	gaaaaatattcaacgccaca tgg ....CCTTTC - - - GTTCTCGAGAGAGC

C

FAD binding motif — *deletion*

MENMFRLMDQDQDLTNNRCIWVNGPVIVGAGPSGLATAACLHEQNVPFVVLERADCIASLWQKRTYDRLKL

HLPKQFCQLPKMPFPEDFPEYPTKRQFIDYLESYATRFEINPKFNECVQTARFDETSGLWRVKTVSKSESTQTEVE

ATG containing motif 1 — *FMO-identify sequence motif* — *NADPH binding motif*

YICRWLVVATGENAERVMPEIDGLSEFSGEVIHACDYKSGEKFAGKKVLVVGCGNSGMEVSLDLANHFAKPSM

VVRSSLHVMPREVMGKSTFELAMKMLRWFPLWLVDKILLVLSWMVLGNIEKYGLKRPEMGPMELKSVKGKT

ATG containing motif 2

PVLDIGAIEKIRLGKINVVPGIKRFNGNKVELVNGEQLDVDSVVLATGYRSNVPYWLQENEFFAKNGFPKTVAD

NNGWKGRTGLYAVGFTRKGLSGASMDAVKIAQDIGSVWQLETKQPTKRSRGSLRRCISQQF

Supplemental Figure S3. CRISPR-Cas9-induced *yuc3* and *yuc8* alleles. A. Schematic overview of the WT and mutant *YUC3* and *YUC8* alleles. Mutant alleles are indicated by *'CR'*. The positions of CRISPR sgRNA protospacer-adjacent motifs (PAM) are indicated by black arrowheads. Open boxes indicate exons, lines indicate untranslated regions (5'/3' UTRs or introns). The stripe-filled box represents a large deletion. DNA insertion sites are indicated by Y-shaped symbols and deletions by vertical lines. The number and position of nucleotide changes are indicated. B. The sequences of wild-type (WT) and mutant *yuc3*^{CR} and *yuc8*^{CR} alleles. The PAM is underlined. Black dots indicate unchanged sequences in between the shown sequences and red dashes indicate deleted sequences. C. Amino acid sequence of the *yuc8*^{CR2} mutant. Protein motifs are indicated in grey and the single amino acid change in red. The FAD and NADPH motifs were annotated using https://www.unipot.org/Q9SVU0. The FMO-identity sequence motif and ATG containing motif were annotated manually (Vallon, 2000; Cao et al., 2019). D. Reduced fertility in the *yuc8*^{CR2} mutant.

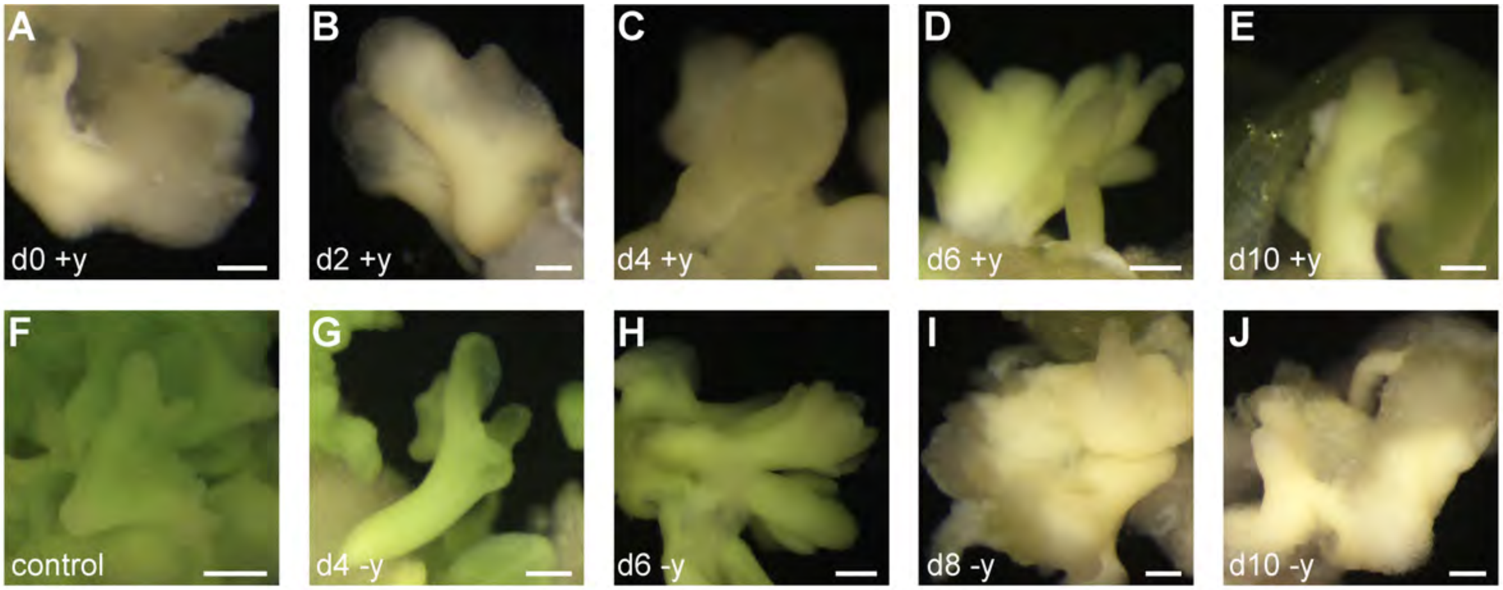

Supplemental Figure S4. Magnified images of 14 day-old DEX and DEX+YUC inhibitor treated *35S:BBM-GR* explants. A-E. DEX-treated *35S:BBM-GR* explants treated with the YUC enzyme inhibitor yucasin (y) on the day of culture, as indicated (day +y). F. DEX-treated *35S:BBM-GR* control. G-J. DEX-treated *35S:BBM-GR* explant treated with yucasin (y) on day 0 of culture followed by removal of yucasin on the indicated day (day -y). 100 µM yucasin was applied in A-E and G-J. For comparison, the same labels (A-J) are used as in Figure 6. The images are magnified versions of the same images shown in Figure 6. Scale bars, 250 µm.

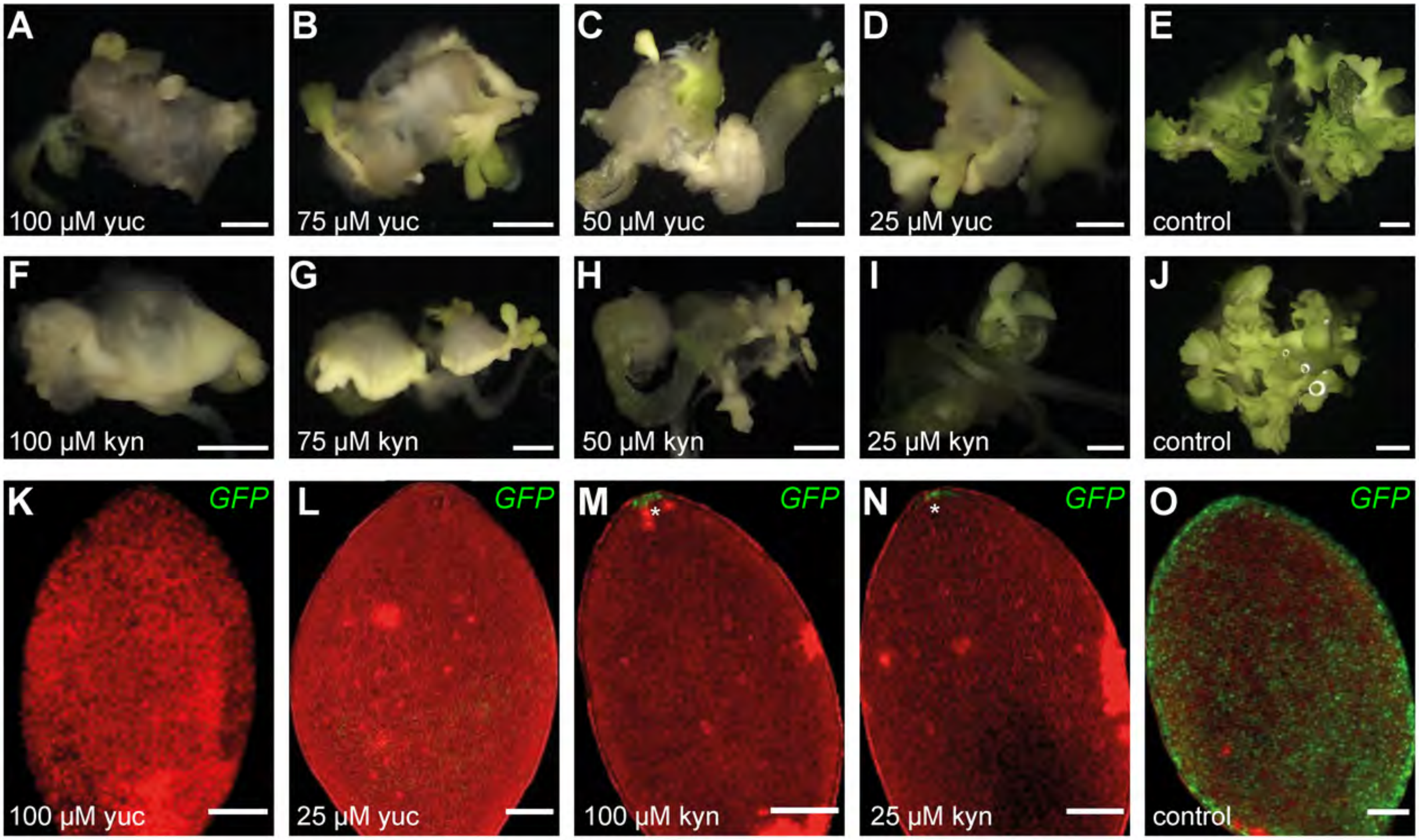

Supplemental Figure S5. Auxin biosynthesis inhibitors block somatic embryo formation and auxin response. *35S:BBM-GR* seeds were grown in the presence of dexamethasone (DEX) with or without the auxin biosynthesis inhibitors yucasin (yuc) or kynurenine (kyn). Images are from 14-day old explants. A-E. Explants from samples treated with DEX and different concentrations of yuc as indicated in each image. Control, DEX only treatment. F-J. Explants from samples treated with DEX and different concentrations of kyn, as indicated in each image. Control, DEX only treatment. K-L. *DR5:GFP* expression in yuc-treated samples. M-N. D*R5:GFP* expression in kyn-treated samples. O. *DR5:GFP* expression in DEX-treated *35S:BBM-GR* control. Images in (K-O) are cotyledons from four day-old explants. Asterisks, autofluorescence. Scale bars: A-J, 1 mm; K-O, 100 µm.

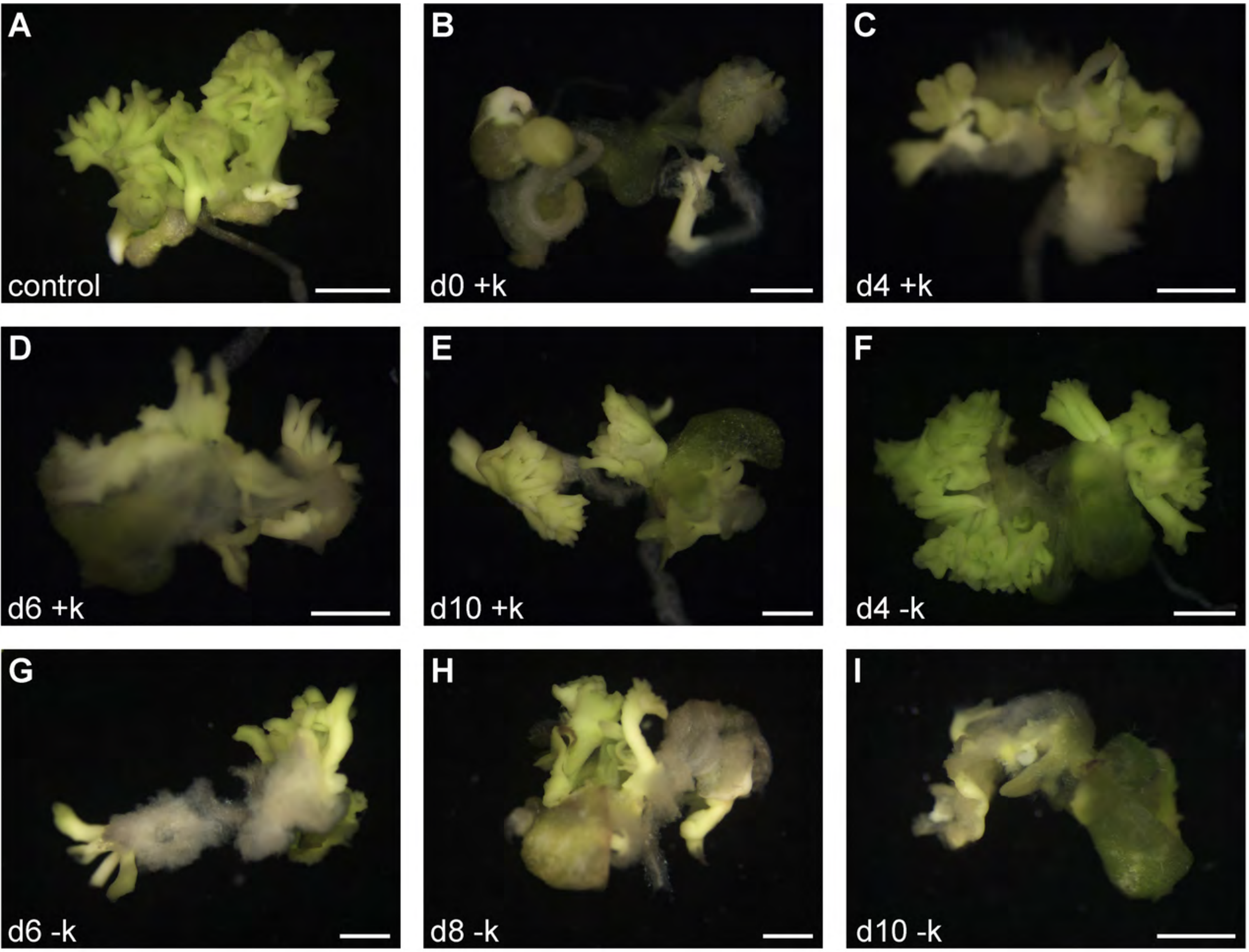

Supplemental Figure S6. TAA1/TAR auxin biosynthesis is required for BABY BOOM (BBM)-mediated somatic embryogenesis. Images of 14 day-old dexamethasone (DEX) (A) and DEX+kynurenine treated (B-I) *35S:BBM-GR* explants. A. DEX-treated *35S:BBM-GR* control. B-E. The auxin biosynthesis inhibitor kynurenine (k) was added on the day of culture as indicated (day +k). F-I. Kynurenine was added on day 0 of culture and removed on the indicated day (day -k). 100 µM kyn was applied. Scale bars, 1 mm.

Supplemental Table S1. Percentage of *35S:BBM-GR WOX2:NLS-3xYFP* seedlings with YFP signal in the cotyledon tip or growth protrusion. All samples were treated with dexamethasone (DEX) from day 0, except for the mock- (ethanol) treated control samples. Yucasin difluorinated analog (YDF) was added and/or removed on the days indicated in the table. From day 8 on, only seedlings with obvious growth protrusions on the cotyledon were examined for *WOX2* expression. The percentage of seedlings with WOX2-YFP expression is shown and was calculated using (number of seedlings with YFP signal/total number of examined seedlings) x 100. ND, not determined.

**Treatment**	**Day 0**	**Day 2**	**Day 4**	**Day 6**	**Day 8**	**Day 10**	**Day 14**	**Day 18**
mock-treated	100% (13/13)	100% (9/9)	ND	ND	ND	ND	ND	ND
DEX	ND	100% (14/14)	80% (16/20)	77% (10/13)	100% (11/11)	ND	22% (2/9)	ND
DEX+YDF (YDF added on day 0)	ND	ND	83% (15/18)	55% (6/11)	47% (8/17)	ND	0% (0/14)	ND
DEX+ YDF (YDF added on day 4)	ND	ND	ND	81% (13/16)	94% (15/16)	ND	0% (0/15)	0% (0/12)
DEX+YDF (YDF added on day 0, removed on day 6)	ND	ND	ND	ND	80% (12/15)	56% (9/16)	0% (0/14)	0% (0/13)

Supplemental Table S2. Single-guide RNAs used for CRISPR-Cas9 mutagenesis and primers used for genotyping CRISPR mutants. The same reverse primer (5’ to 3’: TGTGGTCTCAAGCGTAATGCCAACTTTGTAC) was combined with the indicated primers to amplify the single-guide RNA (sgRNA) backbone from the vector *pICH86966* (Weber et al., 2011; Wang et al., 2019). The CRISPR sgRNA protospacer-adjacent motifs are underlined.

**Target gene**	**Sequence of the forward primer used to amplify sgRNAs (5’ to 3’)**
YUC3	sgRNA1: TGTGGTCTCAATTGCTGCGGGCTTGAAACGTGAGTTTTAGAGCTAGAAATAGCAAG<br>sgRNA2: TGTGGTCTCAATTGAAGACGACTTGATGCAAATGTTTTAGAGCTAGAAATAGCAAG
YUC8	sgRNA1: TGTGGTCTCAATTGCTCTCTCGAGAACAACGAAGTTTTAGAGCTAGAAATAGCAAG<br>sgRNA2: TGTGGTCTCAATTGAAAAATATTCAACGCCACAGTTTTAGAGCTAGAAATAGCAAG
	**Genotyping primers (5’ to 3’)**
YUC3	forward: CGGTCTGAACCGACCATAAT
	reverse: CTTCCATACCGGAGTTTCCA
YUC8	forward: CACCATCGTTGCTCCCTAAT
	reverse: CAAGCCACCGGCAAATATACT

Supplemental Table S3. DNA primers used for RT-qPCR.

Target gene	Primer sequence (5’ to 3’; forward/reverse)
TAA1 (AT1G70560)	TTCGTGGTCAATCTGGATCATGG/ACCACGTATCGTCACCGTACAC
YUC3 (AT1G04610)	ATGGTCGTTCGTAGCGCTGTTC/GCGAGCCAAACGGGCATATACTTC
YUC8 (AT4G28720)	TGCGGTTGGGTTTACGAGGAAAG/GCGATCTTAACCGCGTCCATTG
SAND (AT2G28390)	AACTCTATGCAGCATTTGATCCACT/TGATTGCATATCTTTATCGCCATC
